# A Case of Recurrent Coronary Subclavian Steal Syndrome

**DOI:** 10.7759/cureus.9797

**Published:** 2020-08-17

**Authors:** Mostafa Vasigh, Fidel Martinez, Bashar Ibeche, Syed Huda, Hani Kozman

**Affiliations:** 1 Internal Medicine, State University of New York Upstate Medical University, Syracuse, USA; 2 Cardiology, State University of New York Upstate Medical University, Syracuse, USA; 3 Medicine, State University of New York Upstate Medical University, Syracuse, USA

**Keywords:** recurrent coronary subclavian steal syndrome, cabg, chest pain, percutaneous coronary intervention

## Abstract

Coronary subclavian steal syndrome (CSSS) is one of the rare complications of coronary artery bypass graft surgery (CABG). This phenomenon is a potential complication after left internal mammary artery (LIMA) to left anterior descending artery (LAD) CABG. A proximal stenosis of the left subclavian artery (SA) could cause retrograde flow from LIMA to left SA, which characterizes the mechanism of CSSS. We describe a unique case of recurrent CSSS in a 64-year-old female who presented with one month of exertional dyspnea and acute onset chest pain. She had an extensive coronary artery disease history with CABG 15 years prior to presentation and CSSS treated with left SA stent placement nine years later. She also underwent percutaneous intervention with stents placed in the saphenous vein graft. Although electrocardiogram, cardiac enzymes, and stress test did not show any evidence of acute ischemic changes, perfusion scan detected large areas of partially reversible ischemia. Cardiac catheterization was performed, which showed in-stent restenosis of the left SA and retrograde flow from the LIMA to the left SA indicative of recurrence of CSSS. Left SA arteriogram confirmed in-stent restenosis of the left SA, which was treated with balloon angioplasty and stent placement.

## Introduction

Left internal mammary artery (LIMA) to left anterior descending artery (LAD) coronary artery bypass graft surgery (CABG) is a well-established procedure and has been proven to be beneficial. One rare complication of this procedure is an underdiagnosed phenomenon known as coronary subclavian steal syndrome (CSSS). This happens when there is a substantial occlusion of the left subclavian artery (SA) proximal to the ostia of the LIMA, causing blood to backflow from the LIMA to the left SA to maintain left upper extremity perfusion, especially at times of exertion [1]. If left undiagnosed, this could cause ischemic events and risk of sudden cardiac infarction.

## Case presentation

A 64-year-old female with a past medical history of coronary artery disease, CABG, heart failure with reduced ejection fraction, diabetes mellitus, peripheral arterial disease, and hyperlipidemia, and a significant smoking history presented to the emergency department with exertional dyspnea worsening over the past one month and substernal chest pressure developing acutely on the day of presentation.

The patient had an extensive coronary artery disease history, which included quadruple coronary artery bypass graft surgery with saphenous vein graft (SVG) to right coronary artery (RCA), SVG to the first diagonal artery (SVG-D1), and LIMA-to-LAD T-graft (radial artery) to the posterior lateral/left circumflex artery 15 years ago.. The SVG was previously stented twice due to stenosis. She also had a history of CSSS with retrograde flow from LIMA to left SA nine years after CABG, which was treated with placement of a bare metal stent.

**Figure 1:**
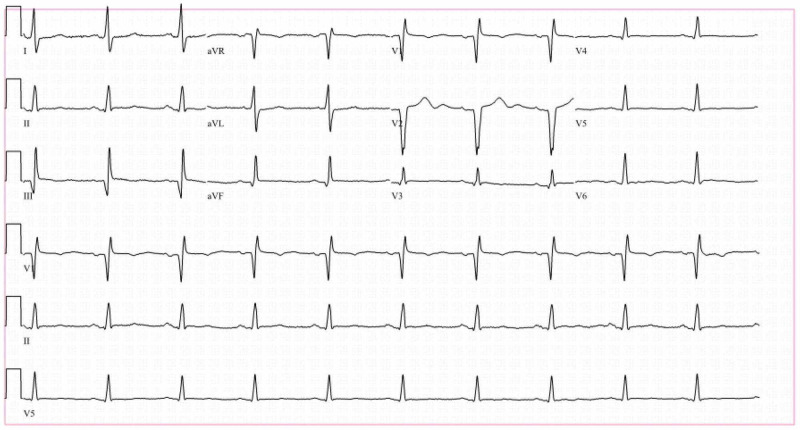
EKG showed atypical RBBB and nonspecific T-wave abnormalities in anterior leads EKG, electrocardiogram; RBBB, right bundle branch block

On arrival to the ED, electrocardiogram (EKG) showed atypical right bundle branch block (RBBB) and nonspecific T-wave abnormalities in the anterior leads with normal troponin T (<0.01 ng/mL) and mildly elevated proBNP ([pro B-type natriuretic peptide] 531 pg/mL) (Figure 1).

A stress EKG was performed, which did not show any evidence of inducible ischemia, but myocardial perfusion scan using technetium 99-m sestamibi detected very large areas of partially reversible ischemia in the inferior and inferolateral walls with stress-induced moderate-to-severe global left ventricular systolic dysfunction and inducible wall motion abnormality.

**Figure 2:**
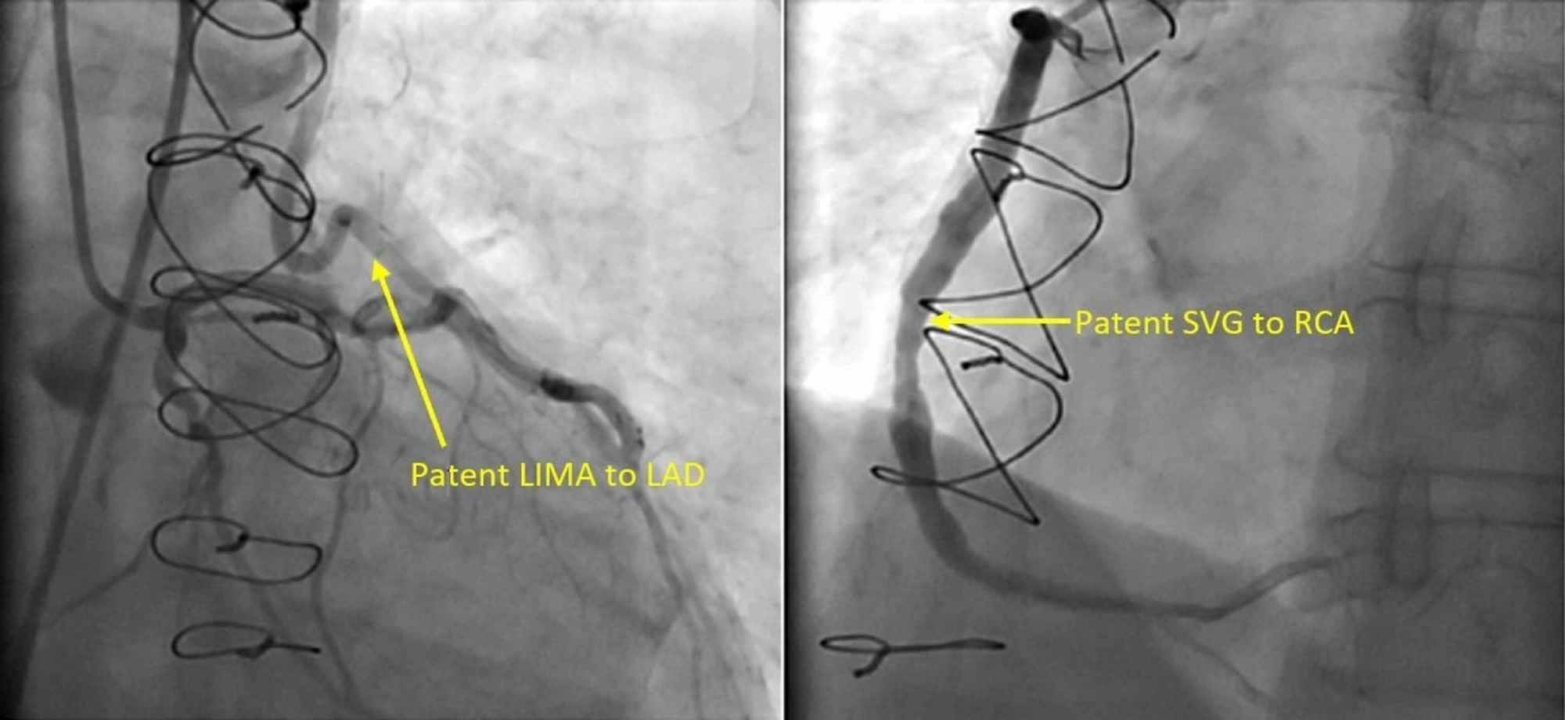
Patent SVG to the RCA and LIMA to LAD grafts SVG, saphenous vein graft; RCA, right coronary artery; LIMA, left internal mammary artery; LAD, left anterior descending artery

Cardiac catheterization was performed, which showed patent LIMA to LAD and SVG to RCA grafts (Figure 2).

**Figure 3:**
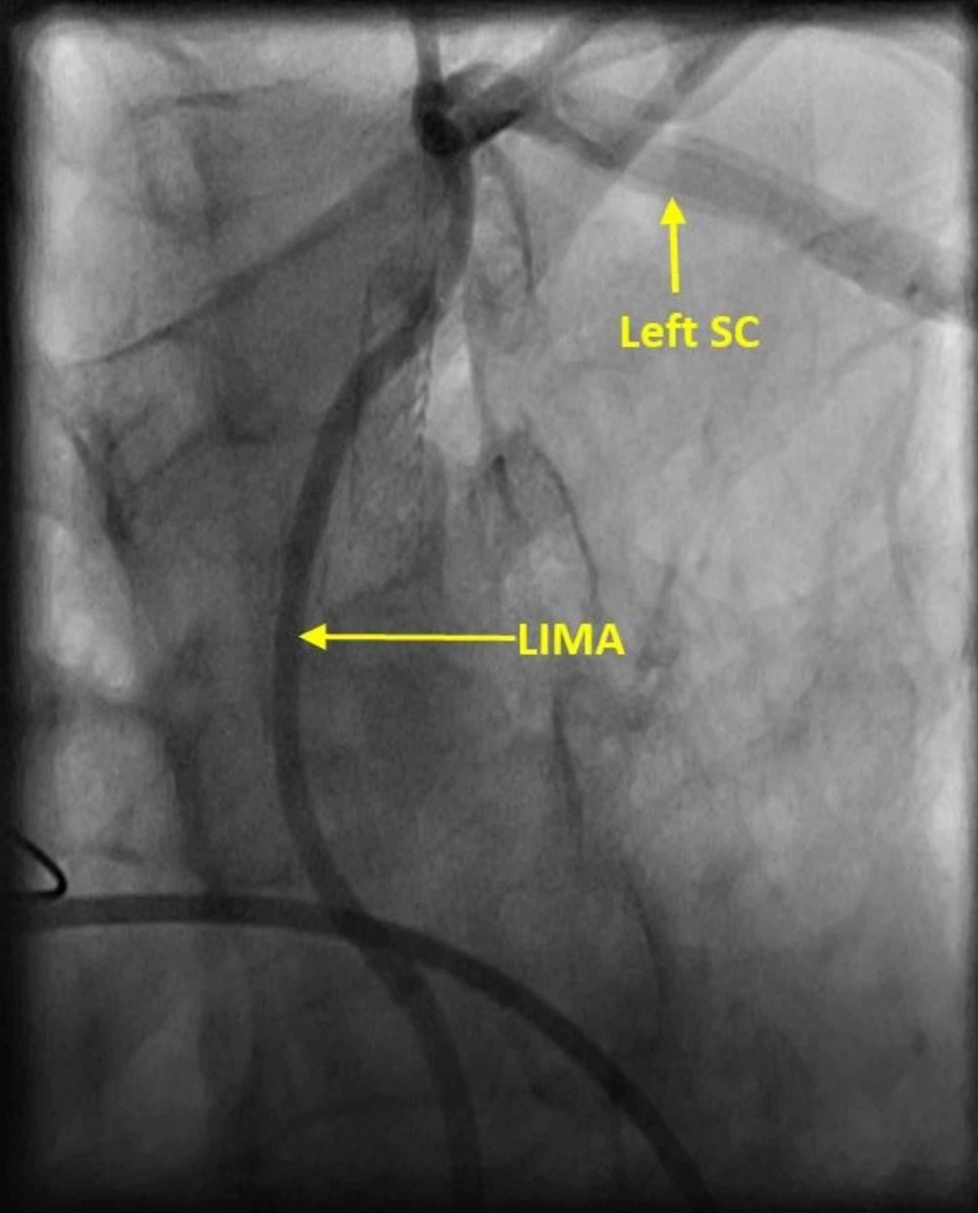
Retrograde flow from the LAD to LIMA and back to the left SA LAD, left anterior descending artery; LIMA, left internal mammary artery; SA, subclavian artery

**Figure 4:**
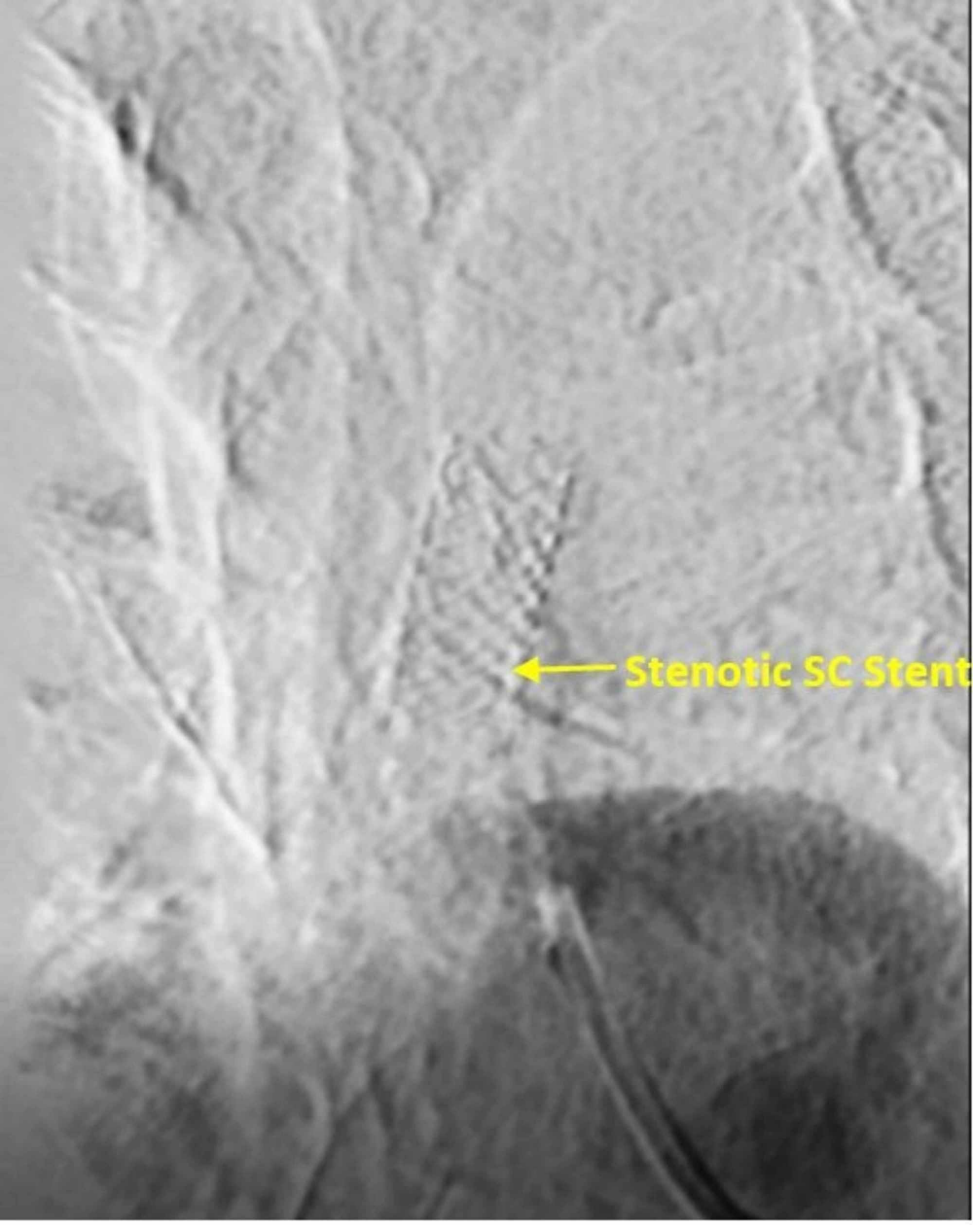
In-stent restenosis of the left SA SA, subclavian artery

But cardiac catheterization also showed retrograde flow from the LAD to LIMA and back to the SA and a 90% in-stent restenosis of the SA (Figures 3, 4).

**Figure 5:**
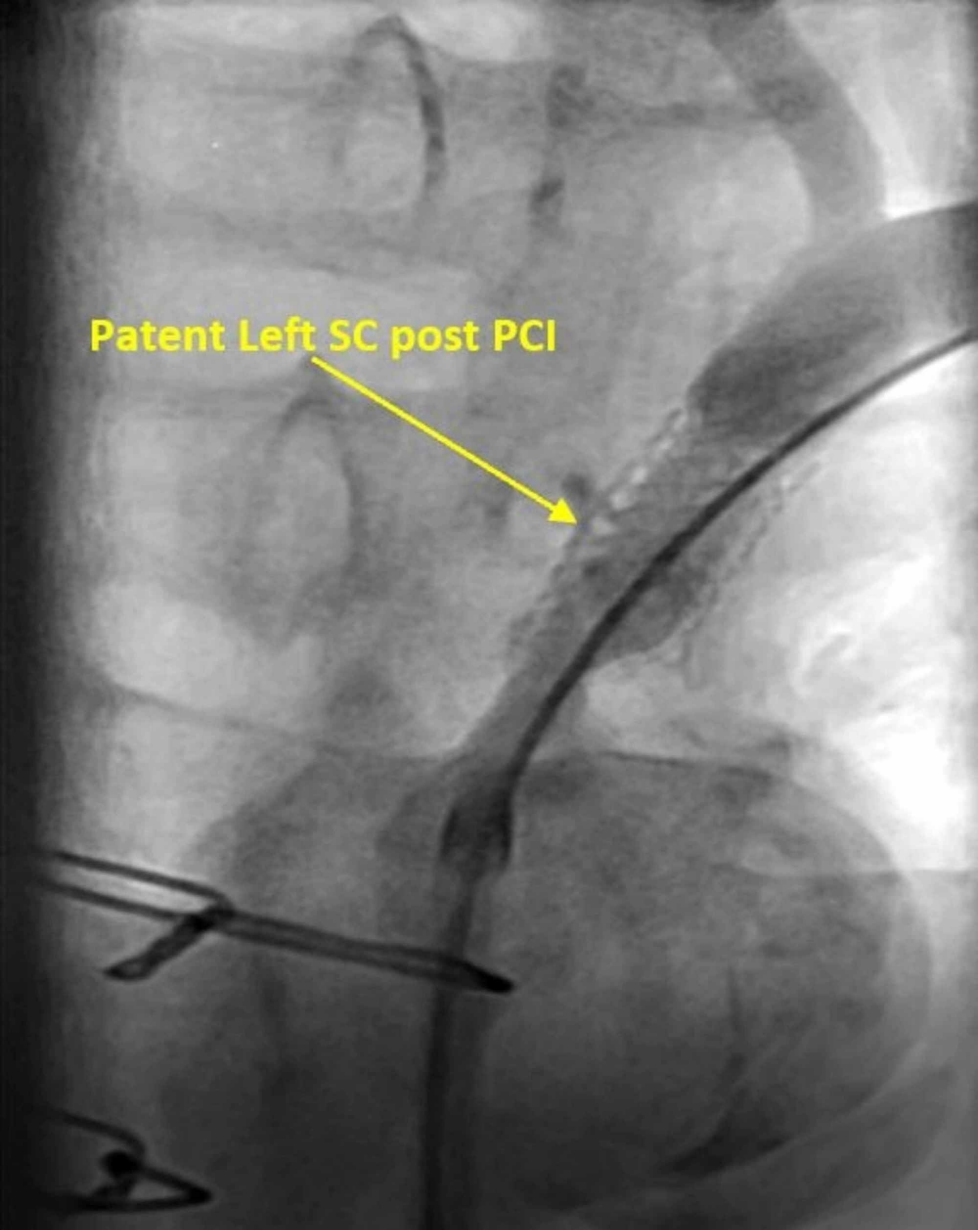
Re-established flow in the left SA after PCI and re-stenting SA, subclavian artery; PCI, percutaneous coronary intervention

Left SA arteriogram was performed, which confirmed left SA in-stent restenosis. The stenosis was ballooned and stented, which re-established flow with 0% stenosis (Figure 5).

After the procedure, the patient was asymptomatic and no complications ensued. She was discharged uneventfully the following day.

## Discussion

CSSS is a rare cause of chest pain post-CABG. Myocardial ischemia is caused by retrograde blood flow from the LIMA to the distal left SA secondary to occlusion in the proximal section of the left SA. CSSS was first described in a case in 1974 [2]. This phenomenon is underdiagnosed, as the most common cause of anginal chest pain in post-CABG patients is atherosclerotic disease of the native coronary arteries or the grafts [3].

Multiple causes have been attributed to the development of CSSS with SA stenosis, with atherosclerotic disease being the most common. Other less common causes include Takayasu arteritis, radiation arteritis, and hemodialysis arteriovenous fistula [4]. The onset of CSSS after CABG has been reported between 2 and 31 years post-procedure. SA calcification risk factors include advanced age, hypertension, diabetes mellitus, and smoking. The highest incidence of SA stenosis has been detected in patients with peripheral arterial disease in other arteries [5].

A positive history of peripheral vascular disease together with an interarm blood pressure difference of more than 20 mm Hg on physical exam are the proposed clinical predictors of SA stenosis [6]. Other important clues to SA stenosis include symptoms of vertebrobasilar insufficiency such as dizziness, ataxia, drop attacks, and upper extremity claudication [7].

CSSS can be diagnosed noninvasively using duplex ultrasound and magnetic resonance angiography, with the presence of blood flow reversal in the vertebral artery being a highly sensitive indicator of SA stenosis in the ipsilateral side. However, the gold standard for the diagnosis of CSSS remains direct subclavian angiography [8].

There are two mainstay treatment approaches to CSSS, endovascular percutaneous transluminal angioplasty (PTA) and peripheral stenting, which are considered firstline treatments compared to the more invasive surgical bypass approach. Despite carrying a shorter hospital stay and less morbidity, PTA has been associated with increased incidence of restenosis and the need for repeat procedure in as many as 28.5% of the cases within a five-year follow-up period [9]. Restenosis rates have been reported to be higher in patients with severe calcification [10]. Other contributing factors to restenosis include peripheral arterial disease, smoking history, and diabetes mellitus.

Surgical bypass approach is generally reserved for patients with a totally occluded SA, when the complete occlusion is near the ostium of the vertebral artery, the length of the lesion is >5 cm, and concomitant brachiocephalic and coronary artery disease is present [8,10]. Despite surgery being a higher risk procedure, it has been shown to be associated with a lower rate of restenosis at 10-year follow-up [7].

The patient presented in this case had a recurrence of CSSS after in-stent restenosis of a previously placed subclavian stent. The patient’s risk factors, including history of peripheral arterial disease, smoking history, and diabetes mellitus, are among the contributing factors resulting in the recurrence of this phenomenon. Reduction of risk factors, better control of comorbid conditions, and routine follow-up can help prevent further episodes and prolong survival of such patients.

## Conclusions

CSSS is a rare complication of CABG, which is mostly seen in patients with significant coronary artery disease, peripheral vascular disease, and multiple risk factors contributing to these conditions. It can recur especially in instances of poor medical follow-up and inadequate risk factor control. Routine follow-up of these patients, elimination of modifiable risk factors, and counselling on lifestyle modification and medical adherence are important in improving morbidity and mortality.
